# Changes in functional connectivity within the fronto-temporal brain network induced by regular and irregular Russian verb production

**DOI:** 10.3389/fnhum.2015.00036

**Published:** 2015-02-18

**Authors:** Maxim Kireev, Natalia Slioussar, Alexander D. Korotkov, Tatiana V. Chernigovskaya, Svyatoslav V. Medvedev

**Affiliations:** ^1^N.P. Bechtereva Institute of the Human Brain, Russian Academy of SciencesSt. Petersburg, Russia; ^2^Faculty of Liberal Arts and Sciences, St. Petersburg State UniversitySt. Petersburg, Russia; ^3^Faculty of Philology, Higher School of EconomicsMoscow, Russia; ^4^Radiological Center of Tyumen Regional Oncology CenterTyumen, Russia

**Keywords:** fMRI, Russian, inflectional morphology, functional connectivity, psycho–physiological interactions, fronto-temporal brain network, dual-route theories, single-route theories

## Abstract

Functional connectivity between brain areas involved in the processing of complex language forms remains largely unexplored. Contributing to the debate about neural mechanisms underlying regular and irregular inflectional morphology processing in the mental lexicon, we conducted an fMRI experiment in which participants generated forms from different types of Russian verbs and nouns as well as from nonce stimuli. The data were subjected to a whole brain voxel-wise analysis of context dependent changes in functional connectivity [the so-called psychophysiological interaction (PPI) analysis]. Unlike previously reported subtractive results that reveal functional segregation between brain areas, PPI provides complementary information showing how these areas are functionally integrated in a particular task. To date, PPI evidence on inflectional morphology has been scarce and only available for inflectionally impoverished English verbs in a same-different judgment task. Using PPI here in conjunction with a production task in an inflectionally rich language, we found that functional connectivity between the left inferior frontal gyrus (LIFG) and bilateral superior temporal gyri (STG) was significantly greater for regular real verbs than for irregular ones. Furthermore, we observed a significant positive covariance between the number of mistakes in irregular real verb trials and the increase in functional connectivity between the LIFG and the right anterior cingulate cortex in these trails, as compared to regular ones. Our results therefore allow for dissociation between regularity and processing difficulty effects. These results, on the one hand, shed new light on the functional interplay within the LIFG-bilateral STG language-related network and, on the other hand, call for partial reconsideration of some of the previous findings while stressing the role of functional temporo-frontal connectivity in complex morphological processes.

## INTRODUCTION

Numerous studies examine morphologically complex forms to compare different models of inflection in the mental lexicon. One of the crucial things they focus on is the distinction between regular and irregular forms. The so-called “dual route” (DR) approach assumes that the former are generated and processed by symbolic rules, while the latter stored in the lexicon, from where they can be retrieved through associative memory mechanisms (e.g., Pinker and Prince, 1988; Pinker, 1991; Marslen-Wilson and Tyler, 1997; Orsolini and Marslen-Wilson, 1997; Clahsen, 1999; Ullman, 2004). According to the “single route” (SR) approach, all forms are computed by a single integrated system that contains no symbolic rules (e.g., Rumelhart and McClelland, 1986; MacWhinney and Leinbach, 1991; Plunkett and Marchman, 1993; Ragnasdóttir et al., 1999; McClelland and Patterson, 2002).

Behavioral studies testing DR and SR approaches analyze a variety of languages, but neuroimaging studies rely primarily on English and German data (e.g., Jaeger et al., 1996; Indefrey et al., 1997; Ullman et al., 1997; Marslen-Wilson and Tyler, 1998; Münte et al., 1999; Newman et al., 1999, 2007; Beretta et al., 2003; Sach et al., 2004; Joanisse and Seidenberg, 2005; Desai et al., 2006; Sahin et al., 2006; Oh et al., 2011). Inflectional morphology in morphologically richer languages like Finnish, Polish, and Arabic was examined in a number of neuroimaging studies (e.g., Lehtonen et al., 2006; Boudelaa et al., 2010; Leminen et al., 2011; Szlachta et al., 2012). However, these studies did not compare regular and irregular forms, focusing on other problems (the distinction between inflectional and derivational morphology, the role of general perceptual and specifically linguistic complexity, etc.).

In the present study, we turned to Russian, a language with rich and diverse morphology, and conducted an fMRI investigation where participants were asked to generate present tense forms from different real and nonce (nonword) verbs and to pluralize real and nonce nouns. Addressing the problem of regularity in a morphologically rich language is important because one can tease apart several factors that are confounded in a language like English (while English definitely has its own advantages with its minimalist system and sharp contrasts between inflectional classes). To give one example, all regular past tense forms are morphologically complex in English, i.e., contain a stem and a suffix (*-ed*), while irregular forms are morphologically simplex. In Russian, all past tense forms are morphologically complex, which gives us an opportunity to find out whether the effects observed in English were due to regularity or to morphological complexity. Other properties of Russian that may be relevant for the debate will be pointed out in Section “A Brief Description of the Russian Verb and Noun Systems.” We opted for a production task because it was used in the majority of neuroimaging studies focusing on regular vs. irregular inflectional morphology.

Experimental data reflecting the localization and the direction of the change in functional activity are reported in Slioussar et al. (2014). In this paper, we present a ROI-whole brain voxel-wise analysis of context dependent changes in functional connectivity [a psychophysiological interaction (PPI) analysis; Gitelman et al., 2003]. The first type of analysis makes it possible to reveal functionally segregated brain areas that change their activity in response to experimental manipulations, while PPI is a measure of functional connectivity, which provides complementary information showing how these segregated brain areas are integrated (Friston, 2011). Although PPI analysis does not make it possible to infer causal relationships, it gives an opportunity to observe how the functional interplay between involved brain regions is changed as a function of the psychological context.

Therefore, we saw PPI analysis as a valuable tool to approach the problem from a new angle, especially given the fact that we found only one previous PPI study of inflectional morphology (Stamatakis et al., 2005). Important similarities and differences between Stamatakis et al.’s (2005) findings and our results offer a novel perspective on our findings from Slioussar et al. (2014), the account proposed by Stamatakis et al. (2005) and a number of problems discussed in other studies.

### A BRIEF DESCRIPTION OF THE RUSSIAN VERB AND NOUN SYSTEMS

The Russian verb system is very complex, and there are several approaches to dividing verbs into classes. According to the one developed in Jakobson (1948), Townsend (1975) and Davidson et al. (1996), Russian has 11 verb classes and several so-called anomalous verbs. Ten classes are identified by their suffixes, while the 11th class has a zero suffix, and is subdivided into subclasses depending on the quality of the root-final consonant [Jakobson (1948) and Townsend (1975) counted them as 13 separate classes].

All verbs have two stems: the present/future tense stem and the past tense stem. Depending on the class, the correlation between them may include truncations or additions of the final consonant or vowel, stress shifts, suffix alternations, alternations of stem vowels, and stem-final consonants. The verb class also determines which set of endings is used in the present and future tense (first and second conjugation types). Usually, the class is unrecoverable from a particular form. For example, *délat*’ ‘to do’ belongs to the AJ class, and its third person plural present tense form is *déla-j-ut* (-*j*- suffix is added, first conjugation type).^1^ *Pisát’* ‘to write’ belongs to the A class, and its third person plural present tense form is *píš-ut* (-*a*- suffix is truncated, first conjugation type, final consonant alternation, stress shift). *Deržát*’ ‘to hold’ belongs to the ZHA class, and its third person plural present tense form is *derž-át* (-*a*- suffix is truncated, second conjugation type).

Verb classes dramatically differ in frequency, and five of them are productive. Thus, there is no single productive pattern that can be applied to any stem irrespective of its phonological characteristics, and no obvious division into regular verbs (RVs) and irregular verbs (IVs) in this system. In our fMRI experiment, we decided to look at the two poles of this system, comparing verbs from the most frequent and productive AJ class to verbs from small unproductive classes (we reasoned that if any differences between these two groups were found, we could compare them to other verbs in subsequent studies). For the sake of brevity, we will further call these groups *regular* and *irregular*.

Russian nouns are inflected for number and case and are classified into different declensions depending on the set of their number and case endings. In many ways, this system is simpler than the system of verb classes. There are only three declensions (plus a group of nouns with adjectival endings, several exceptional cases and a number of uninflected nouns). These declensions differ in frequency, but all three are productive. Usually, the declension can be unambiguously determined from the nominative singular form. Inside every declension there are small groups of nouns with minor irregularities: unusual endings in some forms or stem alternations. For our study we selected a group of nouns that lose the last vowel of the stem in many forms including the nominative plural form (e.g. *koster* ‘fire’ – *kostry*) and a group where the stem never changes, as in the majority of Russian nouns (e.g., *šofer ‘driver’ – šofery*). We will further call the first group *irregular*, although this is a relatively minor irregularity.

### PREVIOUS STUDIES TESTING THE SR AND DR APPROACHES ON RUSSIAN

Behavioral studies testing SR and DR approaches on Russian looked at adult native speakers, L1 and L2 learners and subjects with various neurological and developmental deficits (e.g., Gor and Chernigovskaya, 2001, 2003, 2005; Gor, 2003, 2010; Chernigovskaya et al., 2007; Svistunova, 2008; Gor et al., 2009; Gor and Jackson, 2013). Participants were provided with infinitives or past tense forms of real or nonce verbs and prompted to generate first person singular and third person plural present tense forms. The findings did not unambiguously support either DR or SR approach. For example, on one hand, adults were shown to use the most frequent AJ class pattern as the default one, although Russian has several highly frequent productive verb classes. In particular, it was often applied to nonce verbs irrespective of their morphonological properties. On the other hand, children consecutively overgeneralize several conjugational patterns in the course of acquisition. As a result, the group of authors working on Russian argued that Yang’s (2002) model relying on multiple rules of different status might be better suited to account for their findings. A similar model for Russian was developed by Gor (2003).

Subtractive analysis of the data from the present experiment we reported in Slioussar et al. (2014) is the only fMRI study of Russian inflectional morphology we are aware of. Previous neuroimaging studies arguing for the DR approach, as well as a number of studies that do not directly address the DR vs. SR debate (e.g., Marslen-Wilson and Tyler, 2007; Bozic et al., 2010, 2013; Szlachta et al., 2012), argue that rule-based processing is supported by the fronto-parietal network, particularly by Broca’s area. However, only two fMRI studies comparing regular vs. irregular form production found more activation in Broca’s area for regulars (Dhond et al., 2003; Oh et al., 2011). Increased left IFG activation for regulars was also observed in an fMRI study where the processing of spoken regular and irregular forms was compared in a same-different judgment task (Tyler et al., 2005).

Other fMRI studies report the opposite pattern: Broca’s area was activated more by irregulars (Beretta et al., 2003; de Diego-Balaguer et al., 2006; Desai et al., 2006; Sahin et al., 2006). Two alternative explanations are proposed. Proponents of the DR approach suggest that these results can be explained by conflict monitoring between the regular rule and irregular form or by inhibition of regular rule application (e.g., Sahin et al., 2006). Desai et al. (2006) argue for the SR approach: they conclude that the observed activation differences reflect the greater processing load posed by irregulars, which rely on less frequent inflection patterns than RVs and therefore have greater attentional and response selection demands.

In Slioussar et al. (2014), nonce verbs and nouns were added to the comparison. Participants silently read stimuli and produced aloud particular forms from them. We found that functional activity within the fronto-parietal network was influenced by regularity and lexicality: it was greater for IVs than for regular ones and for nonce verbs than for real ones. We demonstrated that the effects of regularity and lexicality were very similar and concluded that the observed BOLD changes were induced not by (ir)regularity as such, but by the increase of processing load from RV to irregular (IV) to regular nonce verb (RNV) to irregular nonce verbs (INV).

This conclusion was supported by the (RV > B) < (IV > B) < (RNV > B) < (INV > B) parametric contrast, where B is an implicitly modeled baseline, and by behavioral results: the number of mistakes increased from RV to IV to RNV to INV condition. The results for nouns were similar. Only the main effect of regularity did not reach significance in the factorial analysis of fMRI data – presumably, because the only irregular feature we could find for our noun stimuli was rather minor (see A Brief Description of the Russian Verb and Noun Systems).

### A PREVIOUS PPI STUDY OF INFLECTIONAL MORPHOLOGY AND THE PRESENT STUDY

We were only able to find only one PPI study of inflectional morphology (Stamatakis et al., 2005). In this study, functional connectivity between functionally predefined regions of interest (ROIs) located in the left inferior frontal gyrus (LIFG), anterior cingulate cortex (ACC), superior temporal gyrus (STG), and middle temporal gyrus (MTG) was assessed during the same/different judgment task. Stimuli were aurally presented pairs of English words and nonce words, in particular, RV and IV pairs like *jumped – jump* and *thought – think*.

Stamatakis et al. (2005) report a positive influence of LIFG activity on the activity in the left STG/MTG and a modulatory influence of ACC activity on this fronto-temporal connectivity. The former effect did not depend on regularity *per se*, but we know from the subtractive analysis of the data reported in Tyler et al. (2005) that RVs activated the LIFG, bilateral STG and MTG significantly more than irregular ones in this study. The latter effect was significantly stronger for regulars than for irregulars. Stamatakis et al. (2005) believe that these findings indicate greater engagement of the fronto-temporal network in RV processing, with the ACC playing a monitoring role. They conclude: “this reflects the additional processing demands posed by regular inflected forms, requiring modulation of temporal lobe lexical access processes by morphological parsing functions supported by the LIFG” (p. 116).

Undertaking a PPI analysis of our data, we were primarily interested in two things. Firstly, an advantage of this approach is that task-dependent connectivity changes may be detected even when the levels of functional brain activity are not affected by experimental manipulations. We aimed to reveal functional interactions underlying changes in functional activity observed within the LIFG during regular and irregular form production (Slioussar et al., 2014). As we noted above, the increase in LIFG activity in IV trials was explained by the difference in processing load between these two tasks in Slioussar et al. (2014). In principle, this difference could attenuate functional activity changes associated with regularity. Therefore we turned to PPI analysis to find out whether this was indeed the case and to tease apart connectivity changes associated with morphological properties and with cognitive demands.

Secondly, we were interested how our findings would compare to Stamatakis et al.’s (2005) given several important differences in our experiments. First of all, there are obvious differences in the experimental task and in the language used (morphologically poor English vs. morphologically rich Russian). Furthermore, subtractive analyses presented in Tyler et al. (2005) and Slioussar et al. (2014) revealed the opposite results, in particular, the LIFG was more activated by regulars in the first study and by irregulars in the second. Finally, the analyses of behavioral data (the number of mistakes in different conditions) showed that irregular trials were more difficult than regular ones for the participants of our study, while Tyler et al. (2005) reported very similar accuracy rates.

In general, we wanted to see whether the functional connectivity of LIFG would be substantially different during comprehension and production of regular vs. irregular forms (although our task definitely involves a silent reading stage as well). In particular, we expected that if the findings from Stamatakis et al. (2005) are genuine regularity effects, we might be able to replicate them despite all the differences, teasing them apart from processing difficulty effects identified in Slioussar et al. (2014). Foreshadowing the results, this is exactly what we did in the present study.

## MATERIALS AND METHODS

### PARTICIPANTS

Twenty-one healthy subjects participated in the study (13 females, 8 males). All participants were native speakers of Russian, 19–32 years of age, with no history of neurological or psychological disorders. All participants were right-handed, as assessed by the Edinburgh Handedness Inventory (Oldfield, 1971). Subjects were given no information about the specific purpose of the study. All subjects gave their written informed consent prior to the study and were paid for their participation. All procedures were in accordance with the Declaration of Helsinki and were approved by the Ethics Committee of the N.P. Bechtereva Institute of the Human Brain, Russian Academy of Sciences.

### MATERIALS

Materials consisted of eight groups of real and nonce verbs and nouns, illustrated in **Table 1** (a complete list is given in Supplementary Material). The first group of 35 real verbs belonged to the AJ class (RV); the second group contained 35 verbs from several small non-productive classes (IV). Only unprefixed imperfective verbs were used. Two matching groups of 35 nonce verbs (RNVs and INVs) mimicked the general characteristics of the corresponding real verb groups (length and phonological properties of the stem).

**Table 1:** Examples of stimuli in different conditions.

Condition	Presented forms	Correct answers
Regular verbs (RV)	*kivat’* ‘to nod’	*kivaju*
Irregular verbs (IV)	*kolot’* ‘to cleave, to sting’	*kolju*
Regular nonce verbs (RNV)	*vupat’*	*vupaju*
Irregular nonce verbs (INV)	*xorot’*	*xorju*
Regular nouns (RN)	*sokol* ‘falcon’	*sokoly*
Irregular nouns (IN)	*posol* ‘embassador’	*posly*
Regular nonce nouns (RNN)	*mokol*	*mokoly* (and *mokly*)
Irregular nonce nouns (INN)	*fopol*	*foply* (and *fopoly*)

The first group of 35 real nouns had no stem changes (regular nouns, RN), while in the second group the last vowel of the stem was dropped in many forms including the nominative plural form (irregular nouns, IN): e.g., *šofer* ‘driver’ – *šofery* vs. *koster* ‘fire’ – *kostry*. All nouns were masculine, belonged to the first declension and had the nominative plural form ending in *-y*. Two groups of 35 nonce nouns (regular nonce nouns, RNN, and irregular nonce nouns, INN) were created to match two real noun groups. Frequency was balanced for all real stimulus groups using *The Frequency Dictionary of the Modern Russian Language* (Lyashevskaya and Sharoff, 2009). Stimuli in all groups were matched for length (see Supplementary Material).

Vowels are dropped only in a subgroup of noun stems ending in particular vowel and consonant clusters (e.g., *-er, -or, -el, -ol* etc.). We selected stems with such clusters both for irregular and for RN groups so as not to make the former more phonologically homogenous than the latter. Final vowel dropping is usually predictable from the combination of consonants before this vowel and from the position of the stress. However, since stimuli were presented visually, no information about stress was available for nonce nouns, and different nominative plural forms could be licitly derived from them.

### LANGUAGE PROTOCOL AND EXPERIMENTAL fMRI PARADIGM

In total, we had 280 stimuli. Each stimulus was visually presented for 700 ms. Fixation crosses (“xxxxx”) were displayed during inter-stimulus intervals, which varied between 3100 and 3500 ms with a 100 ms step. 140 “null-events” (fixation crosses) were pseudo-randomly intermixed with the stimuli (Friston et al., 1999). The experiment was divided into three consecutive runs with 2–5 min rest between them and was preceded by a short practice run. The first 10 dummy scans of each run were discarded. Stimulus delivery and synchronization with fMRI data acquisition were carried out via the Eloquence fMRI System (*In vivo*) and E-Prime software (version 1.1, Psychology Software Tools Inc., Pittsburgh, PA, USA).

Verbs were presented in the infinitive form, and nouns were presented in the nominative singular form. Subjects were instructed to generate aloud as fast as possible the first person singular present tense form if they saw a verb or the nominative plural form if they saw a noun. All responses were recorded simultaneously with fMRI data acquisition by means of the Persaio MRI Noise Cancelation System (Psychology Software Tools, Inc.). Their correctness was assessed oﬄine. When a participant’s responses were no longer appropriate for the target’s category, the corresponding trials were discarded in the subsequent fMRI analyses.

### MR IMAGING PROTOCOL

Magnetic resonance imaging was performed on a 3 Tesla Philips Achieva scanner. In addition to a scout sequence, participants underwent structural and functional imaging. Structural images were acquired applying a T1-weighted pulse sequence (T1W-3D-FFE; TR = 2.5 ms; TE = 3.1 ms; 30° flip angle) measuring 130 axial slices (field of view, FOV = 240 mm × 240 mm; 256 × 256 scan matrix) of 0.94 mm thickness. Functional images were obtained using an echo planar imaging (EPI) sequence (TE = 35 ms; 90° flip angle; FOV = 208 mm × 208 mm; 128 × 128 scan matrix). Thirty-two continuous 3.5 mm thick axial slices (voxel size = 3 mm × 3 mm × 3.5 mm) covering the entire cerebrum and most of the cerebellum were oriented with reference to the structural image. The images were acquired with a repetition time (TR) of 2000 ms. In order to avoid extensive head motions we used an MR-compatible soft cervical collar.

### CONNECTIVITY ANALYSIS

fMRI data preprocessing included realignment, slice-time correction, spatial normalization, and 8 mm full-width/half-maximum isotropic Gaussian smoothing (for details, see Slioussar et al., 2014). It was carried out using SPM8 software (Wellcome Department of Cognitive Neurology, London, UK). Artifact Detection Toolbox^2^ was used to remove fMRI outliers from the analysis.

**FIGURE 1:**
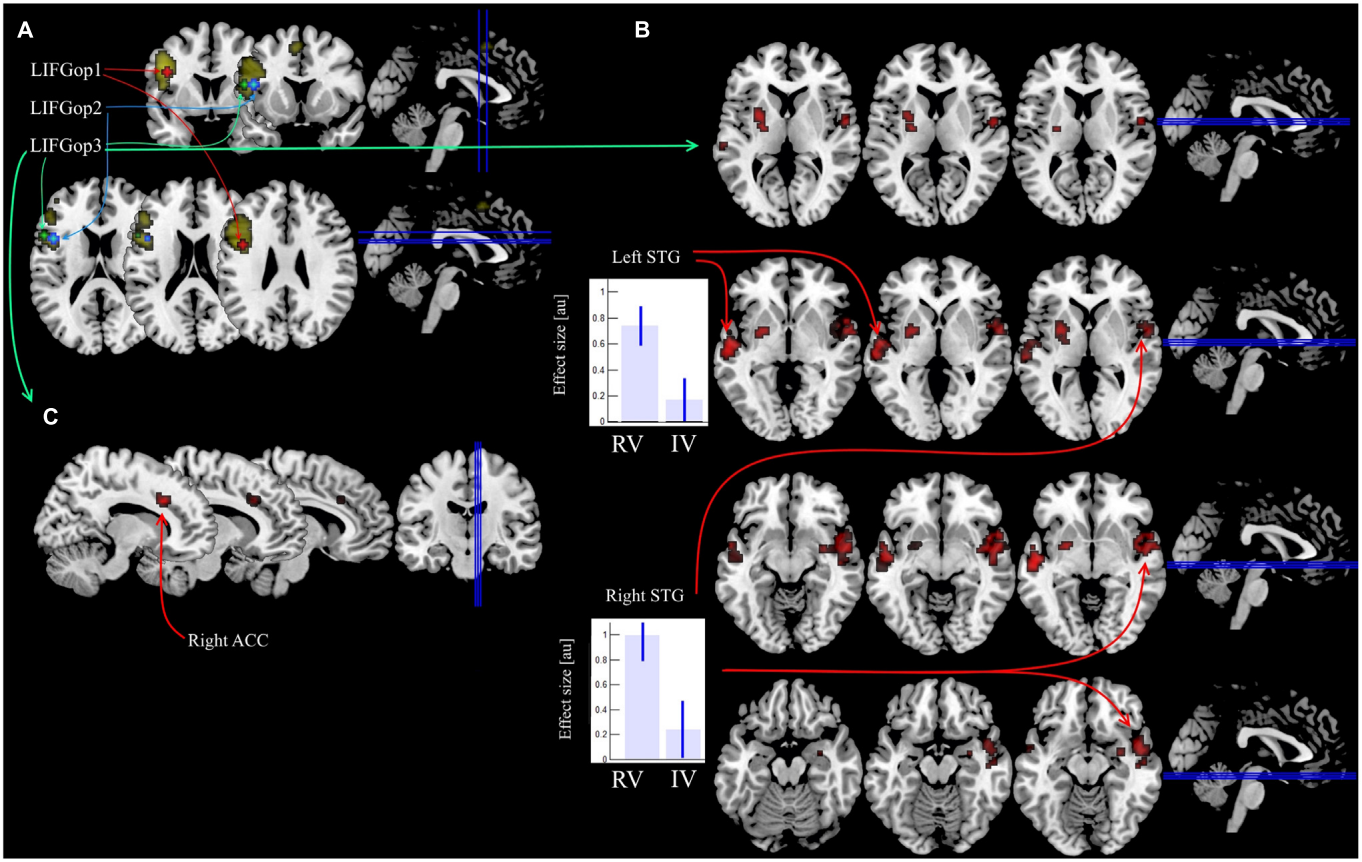
**Location of ROIs in the LIFG and results of PPI analysis. (A)** Three ROIs overlaid on the areas Slioussar et al. (2014) identified as sensitive to the main effect of regularity (regular real and nonce verbs were compared with irregular real and nonce verbs). **(B)** Increase in functional connectivity induced by regular real verb production in the RV > IV comparison for the LIFGop3 seed region. **(C)** Covariance between the number mistakes in irregular real verb production and functional connectivity induced by irregular real verbs in the IV > RV comparison for the LIFGop3 seed region.

During the PPI analysis, ROIs were selected from the cluster in the LIFG, which exhibited greater BOLD values for the production of irregular forms (Slioussar et al., 2014). Three ROIs were created by centering a 4 mm radius sphere in the corresponding local maxima in the opercular part of the LIFG (BA 44, see **Figure 1A**), as defined by the Anatomy toolbox 2.0 (Eickhoff et al., 2005). The analysis of functional connectivity changes was performed between each of the selected ROIs and the remaining voxels of the brain using the generalized PPI toolbox^3^ (McLaren et al., 2012) and included the following steps. First, neuronal activity underlying the observed BOLD changes in every ROI was mathematically estimated (Gitelman et al., 2003). Then the estimated neuronal activity was multiplied by the vectors of each condition’s ON times and convolved with the hemodynamic response function (McLaren et al., 2012; Cisler et al., 2013).

As a result, PPI-regressors corresponding to every experimental trial were created, and the PPI analysis was performed using the general linear model (GLM). Additionally, the GLM included the following nuisance variables: (1) regressors modeling the BOLD signal changes induced by eight experimental conditions and mistake trials (as in the conventional subtractive GLM analysis); (2) head motion parameters and the global mean fMRI outliers; (3) a regressor reflecting the time series of BOLD signal changes within the ROI to exclude context-dependent changes occurring at the hemodynamic level.

To be able to compare our results to those of Stamatakis et al. (2005), we focused on the contrast between regular and irregular *real* word trials in the connectivity analysis, as these authors did. The fronto-temporal connectivity observed by Stamatakis et al. (2005) is most reasonably described as a frontal modulation of lexical access processes, which is obviously not applicable to nonce stimuli. However, the findings from other comparisons are also reported. As in Slioussar et al. (2014), we analyzed verbs and nouns separately rather than putting them together and treating word category as the third factor, primarily because the type of irregularity we were able to find for nouns was very minor compared to what we had in the case of verbs. Thus, RV > IV and IV > RV contrasts of PPI-parameters were estimated with the use of one-sample *t*-tests. Additionally, PPI-parameters for all real and nonce verb trials were analyzed using the ANOVA with two repeated measure factors: lexicality (real vs. nonce) and regularity. The same was done for nouns.

Statistical parametric mappings were computed using the *p* < 0.001 voxel-wise uncorrected threshold. To avoid false positive findings, the FWE *p* < 0.05 correction for multiple comparisons was applied at the cluster level. Since two *t*-tests were calculated for each of the three ROIs, an additional Bonferroni–Holm correction for multiple comparisons was used. The anatomical location of the functional connectivity changes revealed was identified by the Anatomy toolbox. The REX toolbox^4^ was used to demonstrate differences between beta values reflecting functional connectivity changes in the revealed clusters.

### RESULTS

In the RV > IV comparisons, the PPI analysis revealed clusters bilaterally located in the anterior portion of the superior temproral gyri (STG, see **Table 2**; **Figure 1B**). This effect was observed only for the LIFGop3 ROI seed, RV > IV PPI-contrasts for the other two ROI seeds were not significant. Calculating the mean values of PPI-parameters within the obtained clusters pointed to a relative increase in connectivity in RV trials in comparison to IV trials.

**Table 2:** RV > IV PPI-contrast for a ROI seed in the LIFG (BA 44, LIFGop3).

			Peak MNI coordinates		
Brain region	*p*-value	K	X	Y	Z
L. STG (BA 22/21)	0.007*	83	-54	-13	-5
L. putamen	0.028	62	-27	2	4
R. STG/ insula (BA 22/21)	0.0001*	152	54	-11	-1

*Significant clusters after Bonferroni–Holm correction.

*BA, approximate Brodmann’s area; L/R, left/right hemisphere; k, cluster size in voxels; STG, superior temporal gyrus.*

In the IV > RV comparisons, no significant changes in functional connectivity were found for all selected ROI seeds. Since we had concluded in Slioussar et al. (2014) that IV trials were characterized by an increase in processing load in comparison to RV trials, and this conclusion relied not only on neuroimaging, but also on behavioral data (number of mistakes in different conditions), we undertook the following subsidiary analysis to reveal processing load effects. We took the number of mistakes committed by every participant in the IV trials as an individual measure of task difficulty. As we reported in Slioussar et al. (2014), participants made significantly more mistakes in the IV condition than in the RV condition (96 out of 735 vs. 22 out of 735 responses in total, or 13.1% vs. 3.0%, respectively). If we look at each participant separately, the number of mistakes in the IV trials varies from 1 out of 35, or 2.9%, to 9 out of 35, or 25.7%.

Then we submitted IV > RV contrasts of PPI parameters calculated for every participant to the second level group analysis. A one-sample *t*-test, as it is implemented in SPM8, was used with the percentage of mistakes committed by every participant as a variable of interest and estimates of individual IV > RV PPI contrasts as a dependent variable. The results were significant only for one ROI seed, LIFGop3, the same as in the RV > IV PPI analysis above. For this ROI seed, we observed a significant positive covariance between the number of mistakes in the IV trials and the difference in functional connectivity between the LIFG and the right ACC (BA 32; see **Table 3**; **Figure 1C**). Notably, when error rates are low, the difference is negative, i.e., connectivity between the LIFG and the ACC is greater in RV trials. As error rates grow, the difference approaches zero and then becomes positive, i.e., for participants who made more errors than the others, connectivity between the LIFG and the ACC is greater in the IV trials.

**Table 3:** The effect of task difficulty in the IV > RV PPI-contrast for a ROI seed in the LIFG (BA 44, LIFGop3).

			Peak MNI coordinates		
Brain region	*p*-value	*K*	*X*	*Y*	*Z*
R. ACC (BA 32)	0.013*	29	12	20	31

*Significant clusters after Bonferroni-Holm correction.

*ACC, anterior cingulate cortex; BA, approximate Brodmann’s area; L/R, left/right hemisphere; k, cluster size in voxels.*

Comparisons involving real noun stimuli (RN > IN and IN > RN), as well as factorial analyses for verb and noun conditions, did not yield significant results.

## DISCUSSION

We believe that the most noteworthy outcome of the present study is that the connectivity analysis allowed us to dissociate regularity and processing difficulty effects and, as we hope to show below, gain a deeper understanding of their nature. Since we are going to compare our results to Stamatakis et al.’s (2005) and Tyler et al.’s (2005), let us start by highlighting some relevant differences between English and Russian verbs.

Stamatakis et al. (2005) and Tyler et al. (2005) looked at stimulus pairs like *stayed – stay* vs. *taught – teach.* In the regular pairs, the first stimulus was morphologically complex and the second was not, while in irregular pairs, both stimuli were morphologically simple. Obviously, the regular pattern also differs from irregular ones in terms of productivity and type frequency, and it is the morphological default (some authors argue that being the default pattern is a separate property that cannot be reduced to productivity and type frequency, e.g., Clahsen, 1999; Beretta et al., 2003). Behavioral results (error rates) were very similar for regular and irregular sets in this study: 5.1 and 4.3% respectively.

Due to the nature of the Russian language, in our study, all verb stimuli read or produced by the participants were morphologically complex: e.g., *nyr-ja-t’* ‘to dive’ *– nyr-ja-ju* (regular) and *mol-o-t*’ ‘to grind’ *– mel-ju* (irregular). The difference in productivity and type frequency is the same as in English. Finally, there was a difference in error rates in our study, indicating that IVs were more difficult to process. Ideally, all three factors – morphological complexity, regularity and processing difficulty – must be assessed separately and then studied in more detail (for example, to see whether the role of productivity can be dissociated from the role of type frequency etc.). We are infinitely far from this goal now, but arguably, our study lets us make a small step toward it.

Firstly, we observed an increase in functional connectivity between the LIFG and temporal cortex, in particular, the left and right STG, in the RV > IV comparison. Stamatakis et al. (2005) reported similar findings. They saw a positive influence of LIFG activity on the activity in the *left* STG and MTG both for regular and irregular real verb trials. Given that the subtractive analysis of the data reported in Tyler et al. (2005) demonstrated that RVs activated the LIFG, bilateral STG and MTG significantly more than irregular ones in this study, the authors conclude that this indicates greater engagement of the fronto-temporal network in RV processing. Since two PPI studies gave similar results in this case, we suggest that this is an effect of regularity.

Stamatakis et al.’s (2005) study and the present study rely on very different languages: morphologically poor English with a clear-cut distinction between regular and IVs vs. morphologically rich Russian, with numerous verb classes and where the notion of regularity is difficult even to define. Experimental tasks were also different: a same/different judgment task for aurally presented stimuli and a production task for visually presented stimuli (which obviously involves a silent reading stage). The fact that our findings partly replicate Stamatakis et al.’s (2005) despite these major differences shows that the observed regularity effect is indeed robust and has cross-linguistic validity. Moreover, it is present both in comprehension and in production, which we consider good news because no major model addressing the problem of regularity defines this notion differently for production and comprehension. An important advantage of our study that strengthens this result is that we used a ROI-whole brain analysis, i.e., did not predefine the set of regions to be analyzed.

Why did Stamatakis et al. (2005) observe coactivation between the LIFG and the left-lateralized temporal brain network, while in our study, both left and right temporal areas were involved? Given the above-mentioned differences between the two studies, explanations can only be very tentative. This could result from task-related differences: for example, in contrast to passive listening, active word production probably involves self-monitoring associated with bilateral STG activation (e.g., Indefrey, 2011). Alternatively, based on the fact that in Tyler et al. (2005) RVs induced an increase of activation in both *left and right* STG and MTG, one could hypothesize that connectivity changes in the right-lateralized temporal language areas simply did not reach significance in Stamatakis et al. (2005).

In addition to the STG, we also observed an increase in connectivity between the LIFG and the putamen. Since this result did not reach significance after Bonferroni–Holm correction, we will refrain from interpreting it and will only point to some potentially relevant observations in the literature. Numerous studies show that this part of the basal ganglia plays a role in articulation (e.g., Brown et al., 2009, Price, 2010). Some authors also believe that the basal ganglia are part of the system underlying rule-based language processing (e.g., Pinker and Ullman, 2002), but this model is controversial (e.g., Longworth et al., 2005; Macoir et al., 2013). Our observations agree with this model, but could be explained without it. In our stimuli, we matched the length of infinitives, but the present tense forms of some IVs are shorter, so they might require less effort in terms of articulation.

Now let us turn to the results involving the ACC, which did not coincide in the two PPI studies. Stamatakis et al. (2005) found that ACC activity influenced fronto-temporal connectivity, and that the effect was significantly stronger for regulars than for irregulars. They conclude that the ACC plays a monitoring role, “which, in the context of processing real regular inflected words, would reflect greater engagement of an integrated fronto-temporal language system. Morpho-phonological processes, such as the decomposition of regular inflected forms into stems and affixes, may place higher demands on this system, calling on additional resources” (p. 120). Since we did not observe similar results in our study, we hypothesize that this finding is due to the difference in morphological complexity between regular and IVs in English, which is absent in Russian. This hypothesis is very similar to Stamatakis et al.’s (2005) conclusions quoted above, but now we can dissociate morphological complexity from regularity (in the sense of defaultness and/or type frequency).

In our study, we observed covariance between the number of mistakes in the IV trials and functional connectivity changes between the LIFG and the right ACC in the IV vs. RV comparison (see **Figure 1C**). For participants who had low error rates, LIFG–ACC connectivity was greater during RV trials, while for participants who had high error rates, the opposite was true. We believe that we are dealing with two distinct effects here, and that the former is overshadowed by the latter as processing load increases. We do not have a definitive answer as to why LIFG–ACC connectivity may be greater for RVs. Both regular and irregular forms are morphologically complex in Russian and, if there is any rule-based processing system at all, both engage it (infinitival suffixes must be stripped and first person singular endings must be added). However, regular forms might engage this system more than irregular ones: it may also be activated for present tense stem formation. Further research is necessary to test this explanation, but, if it is correct, this would be an argument for the DR approach.

At the same time, LIFG–ACC connectivity increases in IV trials as the processing load they pose grows. This pattern of connectivity changes can be interpreted as a top–down general regulatory effect of the LIFG–ACC interaction, given the fact that the ACC is identified as an important part of the cognitive control network for the detection and resolution of processing conflicts (e.g., Carter and van Veen, 2007; Westerhausen et al., 2010). This effect completely overshadows the one described above when error rates are high. Let us try to formulate more precisely what might be going on. When an irregular form is produced successfully, the stem is simply taken from memory, which is the easiest option for the morphological processing system. But when somebody cannot find the right form and tries to derive it somehow, it is more taxing for the system than dealing with a regular form because the pattern is infrequent and unproductive. In this light, the absence of similar findings in Stamatakis et al.’s (2005) study is expected: different trial types did not differ significantly in terms of processing load in their experiment. This could be due to the fact that Stamatakis et al. (2005) examined comprehension, where one does not have to find or derive any forms. In general, passive comprehension might require more shallow processing than production, and low-status rules or morphological patterns associated with IVs in associative memory might get activated only in the latter case, but not in the former.

Let us briefly comment on the opposite results from Tyler et al. (2005) and Slioussar et al. (2014). The fronto-temporal language-related areas were activated more for irregulars in the former and for irregulars in the latter study. We attributed our findings to processing difficulty (more details above in Section “Previous Studies Testing the SR and DR Approaches on Russian”), while Tyler et al. (2005) explained theirs by regularity. In the light of the Section “Discussion” above, we conclude that in both cases, the increased activity levels might reflect greater engagement of the morphological processing system (this does not contradict the conclusions made in these studies and only clarifies the picture). In English, regular forms rely on it more than irregular ones because the former are morphologically complex, while the latter are simplex and do not require any morphological processing at all. In Russian, all forms are morphologically complex, but when people cannot retrieve an irregular form or try to construct a form from a nonce verb, especially from an INV, the morphological processing system has to work harder.

Now, what do our conclusions mean for the DR and SR approaches? In the SR approach, only the frequency of a morphological pattern really matters. In this respect, regular stimuli had the same properties in both PPI studies, yet the results diverged. The canonical version of the DR approach postulates one default rule and argues that all other forms are stored in memory. Again, *prima facie* this does not predict any differences between regular stimuli in the two studies. One could go on to argue that Russian irregular stimuli must undergo morphological decomposition (at least to get rid of the infinitival affix), and some combination of morphological analysis and memory retrieval processes makes them more difficult than regular stimuli on a certain scale, while English irregular stimuli are the easiest on this scale because no morphological analysis is required at all. Potentially, hybrid models with several rules of different status such as the ones in Yang (2002) are better suited to account for the data. As we mentioned in Section “Previous Studies Testing the SR and DR Approaches on Russian,” such models were proposed for Russian based on the results from behavioral experiments. In any case, it is clear that simplistic views must be discarded.

Further studies are needed to give more definitive answers to the questions above. In particular, the Russian verb system with its numerous classes has much more to offer than what we have used so far. In the present study, we compared verbs from the least frequent unproductive classes to verbs from the most frequent productive AJ class. However, Russian has other highly frequent productive classes. This might allow us to explore the nature of the effects we have observed so far in more detail: what (if anything) would be associated with the morphological default, with productivity, with type frequency, with the complexity of the morphological pattern (e.g., whether it involves stem and suffix alternations etc.)? This might eventually let us figure out what precisely stands behind the regularity effect. Then it will be clear whether it can be accounted for in terms of the DR or SR approach.

Now let us turn to the results for noun stimuli. The fact that the RN > IN comparison gave no significant results is not surprising, given that the main effect of (ir)regularity also did not reach significance for nouns in the factorial analysis in Slioussar et al. (2014). Most probably, this is because the irregular feature we had to select for our noun stimuli was rather minor – the Russian noun system is not very complex in this respect.

Finally, let us look at our data in the context of recent research arguing that fronto-temporal language brain regions are spatially and functionally distinct from the domain-general fronto-parietal multiple demand (MD) system (e.g., Duncan, 2010; Fedorenko et al., 2013; Fedorenko, 2014). In our study, an increase in connectivity between the LIFGop3 region located in one of the language-specific areas and the bilateral STG was driven by linguistic properties of the stimuli (regularity in the sense of defaultness, type frequency and/or productivity). At the same time, we observed how connectivity between this very same region and the right ACC, which is argued to be part of the domain-general cognitive control network, depends on the processing difficulty. As the discussion above shows, the source of this processing difficulty might also be language-specific, namely, it might be a morphological processing difficulty. However, it has an effect on response selection demands, so the cognitive control network must be invoked. In total, in contrast to recent functional connectivity studies arguing for the independence of language-related and domain-general cognitive control systems (Blank et al., 2014), our data demonstrate how these systems can be functionally integrated.

To summarize, the present PPI study allowed us to tease apart processing difficulty and regularity effects in the domain of inflectional morphology. We not only observed the processing difficulty effect we identified earlier in Slioussar et al. (2014), but were also able to find a novel effect of regularity, and gained a better understanding of these two effects by comparing our study to the only other published PPI study of inflectional morphology (Stamatakis et al., 2005). In Slioussar et al. (2014) some regularity-related differences in functional activity could be attenuated by the processing load effect, but the PPI analysis was sensitive enough to reveal such differences in functional connectivity. The present study makes us reconsider some findings from Stamatakis et al. (2005), Slioussar et al. (2014) and several other previous studies.

## Conflict of Interest Statement

The authors declare that the research was conducted in the absence of any commercial or financial relationships that could be construed as a potential conflict of interest.

## Footnotes

1 There are several ways to transliterate Russian words from Cyrillic to Latin alphabet. In this paper, we use the so-called scholarly transliteration system.

2 http://www.nitrc.org/projects/artifact_detect/

3 http://www.nitrc.org/projects/gppi

4 http://www.nitrc.org/projects/rex/

## References

[B1] Beretta A. Campbell C. Carr T. H. Huang J. Schmitt L. M. Christianson K. (2003). An ER-fMRI investigation of morphological inflection in German reveals that the brain makes a distinction between regular and irregular forms. *Brain Lang.* 85 67–92 10.1016/S0093-934X(02)00560-6 12681349

[B2] Blank I. A. Kanwisher N. Fedorenko E. A. (2014). A functional dissociation between language and multiple-demand systems revealed in patterns of BOLD signal fluctuations. *J. Neurophysiol.* 112 1105–1118 10.1152/jn.00884.2013 24872535 PMC4122731

[B3] Boudelaa S. Pulvermüller F. Hauk O. Shtyrov Y. Marslen-Wilson W. D. (2010). Arabic morphology in the neural language system. *J. Cogn. Neurosci.* 22 998–1010 10.1162/jocn.2009.21273 19445607

[B4] Bozic M. Tyler L. K. Ives D. T. Randall B. Marslen-Wilson W. D. (2010). Bihemispheric foundations for human speech comprehension. *Proc. Natl. Acad. Sci. U.S.A.* 107 17439–17444 10.1073/pnas.1000531107 20855587 PMC2951426

[B5] Bozic M. Tyler L. K. Su L. Wingfield C. Marslen-Wilson W. D. (2013). Neurobiological systems for lexical representation and analysis in English. *J. Cogn. Neurosci.* 25 1678–1691 10.1162/jocn-a-00420 23662864

[B6] Brown S. Laird A. R. Pfordresher P. Q. Thelen S. M. (2009). The somatotopy of speech: phonation and articulation in the human motor cortex. *Brain Cogn.* 70 31–41 10.1016/j.bandc.2008.12.006 19162389 PMC2873785

[B7] Carter C. S. van Veen V. (2007). Anterior cingulate cortex and conflict detection: an update of theory and data. *Cogn. Affect. Behav. Neurosci.* 7 367–379 10.3758/CABN.7.4.367 18189010

[B8] Chernigovskaya T. Tkachenko E. Dalbi I. Svistunova T. (2007). “Osobennosti poroždenija glagol’nyx form pacientami s bolezn’ju Alcgejmera (‘Generation of verb forms in Alzheimer’s disease’),” in *Materials of the XXXVI International Philological Conference*, ed. Slepokurova N. (St. Petersburg, FL: St. Petersburg University Press), 73–178.

[B9] Cisler J. M. Bush K. Steele J. S. (2013). A comparison of statistical methods for detecting context-modulated functional connectivity in fMRI. *Neuroimage* 84 1042–1052 10.1016/j.neuroimage.2013.09.018 24055504 PMC4019671

[B10] Clahsen H. (1999). Lexical entries and rules of language: a multidisciplinary study of German inflection. *Behav. Brain Sci.* 22 991–1060 10.1017/S0140525X99002228 11301574

[B11] Davidson D. E. Gor K. S. Lekic M. D. (1996). *Russian: Stage One: Live from Moscow*! Dubuque, IO: Kendall Hunt Publishing Company.

[B12] de Diego-Balaguer R. Rodriguez-Fornells A. Rotte M. Bahlmann J. Heinze H. J. Münte T. F. (2006). Neural circuits subserving the retrieval of stems and grammatical features in regular and irregular verbs. *Hum. Brain Mapp.* 27 874–888 10.1002/hbm.20228 16544328 PMC6871289

[B13] Desai R. Conant L. L. Waldron E. Binder J. R. (2006). fMRI of past tense processing: the effects of phonological complexity and task difficulty. *J. Cogn. Neurosci.* 18 278–297 10.1162/089892906775783633 16494687 PMC1679797

[B14] Dhond R. P. Marinkovic K. Dale A. M. Witzel T. Halgren E. (2003). Spatiotemporal maps of past-tense verb inflection. *Neuroimage* 19 91–100 10.1016/S1053-8119(03)000478 12781729

[B15] Duncan J. (2010). The multiple-demand (MD) system of the primate brain: mental programs for intelligent behavior. *Trends Cogn. Sci.* 14 172–179 10.1016/j.tics.2010.01.004 20171926

[B16] Eickhoff S. B. Stephan K. E. Mohlberg H. Grefkes C. Fink G. R. Amunts K. (2005). A new SPM toolbox for combining probabilistic cytoarchitectonic maps and functional imaging data. *Neuroimage* 25 1325–1335 10.1016/j.neuroimage.2004.12.034 15850749

[B17] Fedorenko E. (2014). The role of domain-general cognitive control in language comprehension. *Front. Psychol.* 5:335 10.3389/fpsyg.2014.00335 PMC4009428 24803909

[B18] Fedorenko E. Duncan J. Kanwisher N. (2013). Broad domain generality in focal regions of frontal and parietal cortex. *Proc. Natl. Acad. Sci. U.S.A.* 110 16616–16621 10.1073/pnas.1315235110 24062451 PMC3799302

[B19] Friston K. J. (2011). Functional and effective connectivity: a review. *Brain Connect.* 1 13–36 10.1089/brain.2011.0008 22432952

[B20] Friston K. J. Zarahn E. Josephs O. Henson R. N. A. Dale A. M. (1999). Stochastic designs in event-related fMRI. *Neuroimage* 619 607–619 10.1006/nimg.1999.0498 10547338

[B21] Gitelman D. R. Penny W. D. Ashburner J. Friston K. J. (2003). Modeling regional and psychophysiologic interactions in fMRI: the importance of hemodynamic deconvolution. *Neuroimage* 19 200–207 10.1016/S1053-8119(03)00058-2 12781739

[B22] Gor K. (2003). *The Rules and Probabilities Model of Native and Second Language Morphological Processing.* St. Petersburg: St. Petersburg University Press.

[B23] Gor K. (2010). Beyond the obvious: do second language learners process inflectional morphology? *Lang. Learn.* 60 1–20 10.1111/j.1467-9922.2009.00549.x

[B24] Gor K. Chernigovskaya T. (2001). “Rules in the processing of Russian verbal morphology,” in *Current Issues in Formal Slavic Linguistics*, eds Zybatow G. Junghanns U. Mehlhorn G. Szucsich L. (Frankfurt am Main: Peter Lang), 528–536

[B25] Gor K. Chernigovskaya T. (2003). Mental Lexicon Structure in L1 and L2 Acquisition: Russian Evidence.

[B26] Gor K. Chernigovskaya T. (2005). “Formal instruction and the acquisition of verbal morphology,” in *Investigation in Instructed Second Language Acquisition*, eds Housen A. Pierrard M. (Berlin: Mouton de Gruyter), 103–139.

[B27] Gor K. Jackson S. (2013). Morphological decomposition and lexical access in a native and second language: a nesting doll effect. *Lang. Cogn. Proc.* 28 1065–1091 10.1080/01690965.2013.776696

[B28] Gor K. Svistunova T. Petrova T. Khrakovskaya M. Chernigovskaya T. (2009). Mental’nyj leksikon pri raspade jazykovoj sistemy u bol’nyx s afaziej: eksperimental’noe issledovanie glagol’noj morfologii (‘The decay of the mental lexicon in aphasia: an experimental study of verb morphology’). *Voprosy Jazykoznanija* 5 3–17.

[B29] Indefrey P. (2011). The spatial and temporal signatures of word production components: a critical update. *Front. Psychol.* 1:255 10.3389/fpsyg.2011.00255 PMC3191502 22016740

[B30] Indefrey P. Brown C. Hagoort P. Herzog H. Sach M. Seitz R. (1997). A PET study of cerebral activation patterns induced by verb inflection. *Neuroimage* 5 S548.

[B31] Jaeger J. J. Lockwood A. H. Kemmerer D. L. Van Valin R. D. Jr. Murphy B. W. (1996). A positron emission tomographic study of regular and irregular verb morphology in English. *Language* 72 451–497 10.2307/416276

[B32] Jakobson R. O. (1948). Russian conjugation. *Word* 4 155–167.

[B33] Joanisse M. F. Seidenberg M. S. (2005). Imaging the past: neural activation in frontal and temporal regions during regular and irregular past-tense processing. *Cogn. Affect. Behav. Neurosci.* 5 282–296 10.3758/CABN.5.3.282 16396090

[B34] Lehtonen M. Vorobyev V. A. Hugdahl K. Tuokkola T. Laine M. (2006). Neural correlates of morphological decomposition in a morphologically rich language: an fMRI study. *Brain Lang.* 98 182–193 10.1016/j.bandl.2006.04.011 16725189

[B35] Leminen A. Leminen M. Lehtonen M. Nevalainen P. Ylinen S. Kimppa L. (2011). Spatiotemporal dynamics of the processing of spoken inflected and derived words: a combined EEG and MEG study. *Front. Hum. Neurosci.* 21:5–66 10.3389/fnhum.2011.00066 PMC3143720 21811451

[B36] Longworth C. E. Keenan S. E. Barker R. A. Marslen-Wilson W. D. Tyler L. K. (2005). The basal ganglia and rule-governed language use: evidence from vascular and degenerative conditions. *Brain* 128 584–596 10.1093/brain/awh387 15659423

[B37] Lyashevskaya O. N. Sharoff S. A. (2009). *Èastotnyj Slovar’ Sovremennogo Russkogo Jazyka (‘The Frequency Dictionary of Modern Russian Language’)*. Moscow: Azbukovnik.

[B38] Macoir J. Fossard M. Mérette C. Langlois M. Chantal S. Auclair-Ouellet N. (2013). The role of basal ganglia in language production: evidence from Parkinson’s disease. *J. Parkinson’s Dis.* 3 393–397 10.3233/JPD-130182 23948988

[B39] MacWhinney B. Leinbach J. (1991). Implementations are not conceptualizations: revising the verb learning model. *Cognition* 40 121–157 10.1016/0010-0277(91)90048-9 1786671

[B40] Marslen-Wilson W. D. Tyler L. K. (1997). Dissociating types of mental computation. *Nature* 387 592–594 10.1038/42456 9177345

[B41] Marslen-Wilson W. D. Tyler L. K. (1998). Rules, representations, and the English past tense. *Trends Cogn. Sci.* 2 428–435 10.1016/S1364-6613(98)01239-X 21227274

[B42] Marslen-Wilson W. D. Tyler L. K. (2007). Morphology, language, and the brain: the decompositional substrate for language comprehension. *Philos. Trans. R. Soc. Lond. B. Biol. Sci.* 362 823–836 10.1098/rstb.2007.2091 17395577 PMC2430000

[B43] McClelland J. L. Patterson K. (2002). Rules or connections in past-tense inflections: what does the evidence rule out? *Trends Cogn. Sci.* 6 465–472 10.1016/S1364-6613(02)01993-9 12457897

[B44] McLaren D. Ries M. Xu G. Johnson S. (2012). A generalized form of context-dependent psychophysiological interactions (gPPI): a comparison to standard approaches. *Neuroimage* 61 1277–1286 10.1016/j.neuroimage.2012.03.068 22484411 PMC3376181

[B45] Münte T. F. Say T. Clahsen H. Schiltz K. Kutas M. (1999). Decomposition of morphologically complex words in English: evidence from event-related brain potentials. *Cogn. Brain Res.* 7 241–253 10.1016/S0926-6410(98)00028-7 9838144

[B46] Newman A. J. Izvorski R. Davis L. Neville H. J. Ullman M. T. (1999). “Distinct electrophysiological patterns in the processing of regular and irregular verbs,” in *Poster presented at the Cognitive Neuroscience Society Annual Meeting,* Washington, DC.

[B47] Newman A. J. Ullman M. T. Pancheva R. Waligura D. L. Neville H. J. (2007). An ERP study of regular and irregular English past tense inflection. *Neuroimage* 34 435–445 10.1016/j.neuroimage.2006.09.007 17070703 PMC1988695

[B48] Oh T. M. Tan K. L. Ng P. Berne Y. I. Graham S. (2011). The past tense debate: is phonological complexity the key to the puzzle? *Neuroimage* 57 271–280 10.1016/j.neuroimage.2011.04.008 21511040

[B49] Oldfield R. C. (1971). The assessment and analysis of handedness: the Edinburgh inventory. *Neuropsychologia* 9 97–113 10.1016/0028-3932(71)90067-4 5146491

[B50] Orsolini M. Marslen-Wilson W. D. (1997). Universals in morphological representation: evidence from Italian. *Lang. Cogn. Proc.* 12 1–47 10.1080/016909697386899

[B51] Pinker S. (1991). Rules of language. *Science* 253 530–535 10.1126/science.1857983 1857983

[B52] Pinker S. Prince A. (1988). On language and connectionism: analysis of a parallel distributed processing model of language acquisition. *Cognition* 28 73–193 10.1016/0010-0277(88)90032-7 2450717

[B53] Pinker S. Ullman M. T. (2002). The past and future of the past tense. *Trends Cogn. Sci.* 6 456–463 10.1016/S1364-6613(02)01990-3 12457895

[B54] Plunkett K. Marchman V. (1993). From rote learning to system building: acquiring verb morphology in children and connectionist nets. *Cognition* 48 21–69 10.1016/0010-0277(93)90057-3 8403834

[B55] Price C. J. (2010). The anatomy of language: a review of 100 fMRI studies published in 2009. *Ann. N. Y. Acad. Sci.* 1191 62–88 10.1111/j.1749-6632.2010.05444.x 20392276

[B56] Ragnasdóttir H. Simonsen H. G. Plunkett K. (1999). The acquisition of past tense morphology in Icelandic and Norwegian children: an experimental study. *J. Child Lang.* 26 577–618 10.1017/S0305000999003918 10603697

[B57] Rumelhart D. McClelland J. (1986). “On learning the past tenses of English verbs,” in *Parallel Distributed Processing: Explorations in the Microstructure of Cognition, Psychological and Biological Models*, Vol. 2 eds Rumelhart D. McClelland J. (Cambridge, MA: MIT Press), 216–271

[B58] Sach M. Seitz R. Indefrey P. (2004). Unified inflectional processing of regular and irregular verbs: a PET study. *Neuroreport* 15 533–537 10.1097/01.wnr.00001 15094518

[B59] Sahin N. Pinker S. Halgren E. (2006). Abstract grammatical processing of nouns and verbs in Broca’s area: evidence from fMRI. *Cortex* 42 540–562 10.1016/S0010-9452(08)70394-0 16881266

[B60] Slioussar N. Kireev M. V. Chernigovskaya T. V. Kataeva G. V. Korotkov A. D. Medvedev S. V. (2014). An ER-fMRI study of Russian inflectional morphology. *Brain Lang.* 130 33–41 10.1016/j.bandl.2014.01.006 24576807

[B61] Stamatakis E. A. Marslen-Wilson W. D. Tyler L. K. Fletcher P. C. (2005). Cingulate control of fronto-temporal integration reflects linguistic demands: a three-way interaction in functional connectivity. *Neuroimage* 28 115–121 10.1016/j.neuroimage.2005.06.012 16023871

[B62] Svistunova T. (2008). *Organizacija Mental’nogo Leksikona. Formirovanie i Raspad Glagol’noj Slovoizmenitel’noj Morfologii: Eksperimental’noe Issledovanie na Material Russkogo Jazyka (‘Mental Lexicon Structure. Development and Decay of the Verbal Inflectional Morphology: An Experimental Study of Russian’)*. Thesis, St. Petersburg State University, St. Petersburg.

[B63] Szlachta Z. Bozic M. Jelowicka A. Marslen-Wilson W. D. (2012). Neurocognitive dimensions of lexical complexity in Polish. *Brain. Lang.* 121 219–225 10.1016/j.bandl.2012.02.007 22541369

[B64] Townsend C. E. (1975). *Russian Word Formation.* Cambridge, MA: Slavica.

[B65] Tyler L. K. Stamatakis E. A. Post B. Randall B. Marslen-Wilson W. D. (2005). Temporal and frontal systems in speech comprehension: an fMRI study of past tense processing. *Neuropsychologia* 43 1963–1974 10.1016/j.neuropsychologia.2005.03.008 16168736

[B66] Ullman M. T. (2004). Contributions of memory circuits to language: the declarative/procedural model. *Cognition* 92 231–270 10.1016/j.cognition.2003.10.008 15037131

[B67] Ullman M. T. Bergida R. O’Craven K. (1997). Distinct fMRI activation patterns for regular and irregular past tense. *Neuroimage* 5 S549.

[B68] Westerhausen R. Moosmann M. Alho K. Belsby S. Hämäläinen H. Medvedev S. (2010). Identification of attention and cognitive control networks in a parametric auditory fMRI study. *Neuropsychologia* 48 2075–2081 10.1016/j.neuropsychologia.2010.03.028 20363236

[B69] Yang C. D. (2002). *Knowledge and Learning in Natural Language.* Oxford: Oxford University Press.

