# Supplementary material for: Changes in functional connectivity within the fronto-temporal brain network induced by regular and irregular Russian verb production
Source: Front Hum Neurosci. 2015 Feb 18;9:36. doi: 10.3389/fnhum.2015.00036 (PMC4332281; doi:10.3389/fnhum.2015.00036)
Supplement: Supplementary file 1 [file Data_Sheet_1.DOCX]

**Appendix**

AJ-class verbs used as test items and analogous nonce verbs^[[1]](#footnote-1)^

|  | **real**  **infinitive** | **present tense**  **1SG form** | **length** | **F^[[2]](#footnote-2)^** | **nonce**  **infinitive** | **present tense**  **1SG form^[[3]](#footnote-3)^** | **length** |
| --- | --- | --- | --- | --- | --- | --- | --- |
| 1 | čitat' | čitaju | 6 | 315.5 | atat' | ataju | 6 |
| 2 | igrat' | igraju | 6 | 249.6 | betat' | betaju | 6 |
| 3 | mešat' | mešaju | 6 | 116.0 | brijat' | brijaju | 6 |
| 4 | želat' | želaju | 6 | 115.6 | vogat' | vogaju | 6 |
| 5 | padat' | padaju | 6 | 107.8 | vupat' | vupaju | 6 |
| 6 | terjat' | terjaju | 6 | 79.2 | gemat' | gemaju | 6 |
| 7 | guljat' | guljaju | 6 | 70.9 | gyrjat' | gyrjaju | 6 |
| 8 | letat' | letaju | 6 | 66.3 | dagat' | dagaju | 6 |
| 9 | menjat' | menjaju | 6 | 58.7 | dopat' | dopaju | 6 |
| 10 | sijat' | sijaju | 5 | 45.9 | žitat' | žitaju | 5 |
| 11 | šagat' | šagaju | 6 | 44.4 | zenjat' | zenjaju | 6 |
| 12 | lomat' | lomaju | 6 | 40.5 | zotat' | zotaju | 6 |
| 13 | kivat' | kivaju | 6 | 39.1 | zuljat' | zuljaju | 6 |
| 14 | pugat' | pugaju | 6 | 31.9 | imlat' | imlaju | 6 |
| 15 | rugat' | rugaju | 6 | 29.5 | ifat' | ifaju | 6 |
| 16 | kušat' | kušaju | 6 | 25.7 | ketat' | ketaju | 6 |
| 17 | kidat' | kidaju | 6 | 22.9 | kibat' | kibaju | 6 |
| 18 | sažat' | sažaju | 6 | 22.5 | lubat' | lubaju | 6 |
| 19 | kopat' | kopaju | 6 | 19.6 | madat' | madaju | 6 |
| 20 | vešat' | vešaju | 6 | 19.0 | mijat' | mijaju | 6 |
| 21 | rydat' | rydaju | 6 | 18.3 | mjapat' | mjapaju | 6 |
| 22 | vlijat' | vlijaju | 6 | 17.9 | nalat' | nalaju | 6 |
| 23 | migat' | migaju | 6 | 17.4 | nydat' | nydaju | 6 |
| 24 | gadat' | gadaju | 6 | 17.1 | ozat' | ozaju | 6 |
| 25 | topat' | topaju | 6 | 17.0 | pelat' | pelaju | 6 |
| 26 | putat' | putaju | 6 | 16.7 | ruvat' | ruvaju | 6 |
| 27 | ronjat' | ronjaju | 6 | 15.2 | somat' | somaju | 6 |
| 28 | motat' | motaju | 6 | 14.8 | tonjat' | tonjaju | 6 |
| 29 | zevat' | zevaju | 6 | 13.8 | fažat' | fažaju | 6 |
| 30 | kapat' | kapaju | 6 | 13.3 | ferjat' | ferjaju | 6 |
| 31 | kusat' | kusaju | 6 | 12.7 | xipat' | xipaju | 6 |
| 32 | nyrjat' | nyrjaju | 6 | 9.6 | xutat' | xutaju | 6 |
| 33 | axat' | axaju | 5 | 4.8 | cadat' | cadaju | 5 |
| 34 | oxat' | oxaju | 5 | 4.7 | čevat' | čevaju | 5 |
| 35 | ikat' | ikaju | 5 | 3.6 | šulat' | šulaju | 5 |
| **Average** |  |  | **5.9** | **49.1** |  |  | **5.9** |

Verbs from small non-productive classes used as test items and analogous nonce verbs

|  | **real**  **infinitive^[[4]](#footnote-4)^** | **present tense**  **1SG form** | **length** | **F** | **nonce**  **infinitive** | **present tense**  **1SG form^[[5]](#footnote-5)^** | **length** |
| --- | --- | --- | --- | --- | --- | --- | --- |
| 1 | brat' | beru | 5 | 302.3 | basti | basu, bastu | 6 |
| 2 | spat' | splju | 5 | 246.2 | bezt' | bezu | 6 |
| 3 | bežat' | begu | 6 | 221.8 | bryt' | broju, bryvu | 6 |
| 4 | nesti | nesu | 5 | 124.1 | vlyzt' | vlyzu | 6 |
| 5 | kolot' | kolju | 6 | 112.3 | vrjast' | vrjadu, vrjanu | 6 |
| 6 | lezt' | lezu | 5 | 78.7 | gesti | gesu, getu, gedu, gebu | 6 |
| 7 | rasti | rastu | 5 | 71.7 | glyt' | gloju, glyvu | 6 |
| 8 | vrat' | vru | 5 | 69.8 | dlesti | dlesu, dletu, dledu, dlebu | 6 |
| 9 | plyt' | plyvu | 5 | 55.7 | dljast' | dljadu, dljanu | 5 |
| 10 | gnat' | gonju | 5 | 49.1 | dorot' | dorju | 6 |
| 11 | polzti | polzu | 6 | 48.2 | žasti | žasu, žastu | 6 |
| 12 | klast' | kladu | 6 | 44.4 | žlyt' | žloju, žlyvu | 6 |
| 13 | rvat' | rvu | 5 | 38.4 | zgat' | zgu | 6 |
| 14 | trjasti | trjasu | 6 | 31.2 | koloč' | koloku, kolku | 6 |
| 15 | revet' | revu | 6 | 27.0 | krat' | kru | 6 |
| 16 | bresti | bredu | 6 | 19.0 | krjasti | krjasu, krjatu, krjadu | 6 |
| 17 | lgat' | lgu | 5 | 16.5 | lpat' | lpu | 6 |
| 18 | teret' | tru | 6 | 14.8 | mkat' | mku | 6 |
| 19 | gryzt' | gryzu | 6 | 13.2 | mljast' | mljadu, mljanu | 6 |
| 20 | cvesti | cvetu | 6 | 10.8 | mresti | mresu, mretu, mredu, mrebu | 6 |
| 21 | gresti | grebu | 6 | 9.5 | nolot' | nolju, nelju | 6 |
| 22 | drat' | deru | 5 | 9.2 | nrast' | nradu | 6 |
| 23 | krast' | kradu | 6 | 7.3 | prast' | pradu | 6 |
| 24 | porot' | porju | 6 | 7.3 | resti | resu, retu, redu, rebu | 6 |
| 25 | plesti | pletu | 6 | 6.6 | sulzti | sulzu | 6 |
| 26 | kljast' | kljanu | 6 | 4.9 | tlast' | tladu | 6 |
| 27 | molot' | melju | 6 | 4.8 | tolot' | tolju, telju | 6 |
| 28 | pasti | pasu | 5 | 4.1 | flat' | flu | 6 |
| 29 | brit' | breju | 5 | 4.0 | xnesti | xnesu, xnetu, xnedu, xnebu | 6 |
| 30 | kryt' | kroju | 5 | 4.0 | xorot' | xorju | 6 |
| 31 | mesti | metu | 5 | 3.1 | cvat' | cvu | 6 |
| 32 | polot' | polju | 6 | 1.8 | čvast' | čvadu | 5 |
| 33 | tkat' | tku | 5 | 1.8 | člyt' | čloju, člyvu | 5 |
| 34 | prjast' | prjadu | 6 | 1.0 | švat' | švu | 5 |
| 35 | toloč' | tolku | 6 | 1.0 | šolot' | šolju, šelju | 5 |
| **Average** |  |  | **5.5** | **47.6** |  |  | **5.5** |

Nouns without vowel dropping in the Nom Pl form used as test items and nonce nouns created on their basis

|  | **real**  **Nom.Sg form** | **Nom.Pl**  **form** | **length** | **F** | **nonce**  **Nom.Sg form** | **Nom.Pl**  **form^[[6]](#footnote-6)^** | **length** |
| --- | --- | --- | --- | --- | --- | --- | --- |
| 1 | otvet | otvety | 5 | 229.7 | azen' | azeni | 5 |
| 2 | avtor | avtory | 5 | 160.6 | akrok | akroki | 5 |
| 3 | sovet | sovety | 5 | 131.3 | ampor | ampory | 5 |
| 4 | primer | primery | 6 | 122.1 | amrek | amreki | 5 |
| 5 | otdel | otdely | 5 | 96.4 | bazdol | bazdoly | 6 |
| 6 | bilet | bilety | 5 | 95.4 | bepret | beprety | 6 |
| 7 | razmer | razmery | 6 | 86.6 | buzlec | buzlecy | 6 |
| 8 | akter | aktery | 5 | 81.2 | vimgol | vimgoly | 6 |
| 9 | šofer | šofery | 5 | 77.5 | vodrom | vodromy | 6 |
| 10 | sekret | sekrety | 6 | 53.9 | goslec | goslecy | 6 |
| 11 | al'bom | al'bomy | 6 | 45.3 | dantor | dantory | 6 |
| 12 | pribor | pribory | 6 | 44.0 | žongel | žongely | 6 |
| 13 | lider | lidery | 5 | 43.2 | zider | zidery | 5 |
| 14 | olen' | oleni | 5 | 38.1 | zjugnet | zjugnety | 6 |
| 15 | faktor | faktory | 6 | 36.9 | ixter | ixtery | 5 |
| 16 | kuznec | kuznecy | 6 | 35.7 | kvobel | kvobely | 6 |
| 17 | bjudžet | bjudžety | 6 | 33.1 | kižet | kižety | 5 |
| 18 | diplom | diplomy | 6 | 22.0 | lodmek | lodmeki | 6 |
| 19 | simvol | simvoly | 6 | 21.5 | mokol | mokoly | 5 |
| 20 | igrok | igroki | 5 | 20.0 | obtet | obtety | 5 |
| 21 | razdel | razdely | 6 | 19.8 | okmel | okmely | 5 |
| 22 | porok | poroki | 5 | 19.5 | pjuden' | pjudeni | 6 |
| 23 | podlec | podlecy | 6 | 17.1 | sakver | sakvery | 6 |
| 24 | prorok | proroki | 6 | 16.7 | segrec | segrecy | 6 |
| 25 | uprek | upreki | 5 | 16.1 | trogok | trogoki | 6 |
| 26 | mudrec | mudrecy | 6 | 14.7 | tubrec | tubrecy | 6 |
| 27 | sokol | sokoly | 5 | 13.2 | ul'gom | ul'gomy | 6 |
| 28 | beglec | beglecy | 6 | 13.1 | flider | flidery | 6 |
| 29 | mongol | mongoly | 6 | 8.6 | fozer | fozery | 5 |
| 30 | pogrom | pogromy | 6 | 5.9 | forok | foroki | 5 |
| 31 | probel | probely | 6 | 3.0 | xivlom | xivlomy | 6 |
| 32 | tjulen' | tjuleni | 6 | 2.3 | xoret | xorety | 5 |
| 33 | bujvol | bujvoly | 6 | 2.2 | čedok | čedoki | 5 |
| 34 | sedok | sedoki | 5 | 1.8 | šajvol | šajvoly | 6 |
| 35 | podtek | podteki | 6 | 1.4 | šlipor | šlipory | 6 |
| **Average** |  |  | **5.6** | **46.6** |  |  | **5.6** |

Nouns with vowel dropping in the Nom Pl form used as test items and nonce nouns created on their basis

|  | **real**  **Nom.Sg form** | **Nom.Pl**  **form** | **length** | **F** | **nonce**  **Nom.Sg form** | **Nom.Pl**  **form** | **length** |
| --- | --- | --- | --- | --- | --- | --- | --- |
| 1 | paren' | parni | 6 | 258.7 | bozec | bozcy | 6 |
| 2 | kamen' | kamni | 6 | 209.8 | bonot' | bonti | 6 |
| 3 | veter | vetry | 5 | 209.2 | brenec | brency | 6 |
| 4 | zvonok | zvonki | 6 | 80.1 | vanel | vanly | 6 |
| 5 | koster | kostry | 6 | 79.8 | veltok | veltki | 6 |
| 6 | dvorec | dvorcy | 6 | 76.2 | vrjumok | vrjumki | 5 |
| 7 | spisok | spiski | 6 | 71.7 | golter | goltry | 6 |
| 8 | platok | platki | 6 | 64.8 | gronok | gronki | 5 |
| 9 | lokot' | lokti | 6 | 63.4 | dvegor | dvegry | 6 |
| 10 | remen' | remni | 6 | 53.8 | drubok | drubki | 6 |
| 11 | tanec | tancy | 5 | 47.2 | žarek | žarki | 6 |
| 12 | kozel | kozly | 5 | 44.4 | žener | ženry | 5 |
| 13 | kover | kovry | 5 | 43.3 | zvarec | zvarcy | 6 |
| 14 | glotok | glotki | 6 | 39.1 | zvorok | zvorki | 6 |
| 15 | nosok | noski | 5 | 38.1 | znisok | zniski | 5 |
| 16 | nogot' | nogti | 6 | 36.7 | ketol | ketly | 5 |
| 17 | krjučok | krjučki | 6 | 26.8 | kilec | kil'cy | 5 |
| 18 | kotel | kotly | 5 | 26.3 | ktitok | ktitki | 6 |
| 19 | xrebet | xrebty | 6 | 22.2 | mnatok | mnatki | 6 |
| 20 | žilec | žil'cy | 5 | 21.7 | mogok | mogki | 5 |
| 21 | značok | znački | 6 | 19.8 | numen' | numni | 6 |
| 22 | stanok | stanki | 6 | 18.4 | psorec | psorcy | 6 |
| 23 | larek | lar'ki | 5 | 17.7 | pulor | pulry | 5 |
| 24 | posol | posly | 5 | 17.1 | samen' | samni | 6 |
| 25 | bugor | bugry | 5 | 15.1 | slanok | slanki | 6 |
| 26 | tvorec | tvorcy | 6 | 12.4 | spačok | spački | 6 |
| 27 | klubok | klubki | 6 | 12.2 | tvalom | tvalmy | 6 |
| 28 | starec | starcy | 6 | 11.7 | tvurok | tvurki | 5 |
| 29 | šnurok | šnurki | 6 | 9.0 | tovot' | tovti | 6 |
| 30 | želtok | želtki | 6 | 2.3 | tosel | tosly | 6 |
| 31 | slitok | slitki | 6 | 2.1 | fopol | foply | 6 |
| 32 | grebec | grebcy | 6 | 2.0 | frevet | frevty | 6 |
| 33 | svekor | svekry | 6 | 1.7 | xemen' | xemni | 6 |
| 34 | psalom | psalmy | 6 | 1.2 | čoner | čonry | 5 |
| 35 | ščegol | ščegly | 5 | 1.2 | šnorec | šnorcy | 6 |
| **Average** |  |  | **5.7** | **47.3** |  |  | **5.7** |

1. Here and below, nonce words mimic the general characteristics of the corresponding real word group, but we tried to avoid close resemblance to particular real words. So one-to-one pairing between real and nonce stimuli cannot be made, and the latter are listed in the alphabetical order (according to the Russian alphabet). [↑](#footnote-ref-1)
2. Frequencies (in instances per million) are taken from (Lyashevskaya & Sharoff, 2009). [↑](#footnote-ref-2)
3. The A class model can also be applied to these nonce verbs (e.g. *atat’ – aču*), and a couple of them are compatible with the ZHA class (e.g. *fažat' – fažu*).There were very few such answers in our experiment, and we discarded them. [↑](#footnote-ref-3)
4. It can be noted that a couple of verbs end in *-at’* like AJ class verbs, but have non-syllabic stems. [↑](#footnote-ref-4)
5. Here, if a nonce verb is compatible with several models, all possible forms are listed because in our experiment, they all belong to the same category: to small non-productive classes. [↑](#footnote-ref-5)
6. Dividing nonce nouns in two groups is often arbitrary because most of them are compatible with both models used in the experiment: the last vowel in the stem can be dropped or left in their Nom.Pl forms. [↑](#footnote-ref-6)
